# Beyond metacognition: The dominant role of the general factor of personality in learning adaptation

**DOI:** 10.1016/j.heliyon.2024.e35147

**Published:** 2024-07-25

**Authors:** Peiqian Wu

**Affiliations:** School of Educational Science, Anhui Normal University, Beijing Road No.2, 241000, Wuhu, Anhui, China

**Keywords:** Metacognition, General factor of personality, Ego-resiliency, Learning adaptation, Incremental validity

## Abstract

The notions of metacognition and ego-resilienc seem to commonly represent an ability to adaptively adjust self-control to fit the requirements of environments. The latter presents a general mechanism of adaptive adjustment while the former presents a specific example of learning activity. As ego-resiliency was almost fully indicated by the General Factor of Personality (GFP) as the literature suggested, the present study tested the relationship between the GFP and metacognition and then compared their influences on learning adaptation. As found, the GFP highly overlapped with overall metacognition (*r* = 0.69). Within the three dimensions of metacognition, metacognitive skills correlated with the GFP much higher than metacognitive knowledge and experience, suggesting that the GFP is more linked with the application of metacognition in learning. By comparison, the GFP displayed much higher correlations with metacognitive skills and experience as well as learning adaptation than any Big Five traits, showing its incremental power in correlation with those variables. More importantly, in the mediation model, the GFP was found to be the root reason for the outcomes of learning adaptation and accounted for most of the impact of metacognition on learning adaptation. With these findings, the limitations of the present study and suggestions for future studies were discussed.

## 1 Introduction

Educational psychology, the scientific examination of how individuals acquire knowledge, explores a vast array of topics including teaching techniques, pedagogical processes, and individual learning distinctions [1]. This field investigates cognitive, emotional, behavioral, and social aspects that impact learning processes, leveraging this insight to devise strategies that empower students to thrive academically [2,3].

Research has found a number of influential factors in learning, including personal and environmental variables. For example, physiological factors such as sense perception, physical health, fatigue, time and day of learning, food and drink, age, and atmospheric conditions can all influence learning [4]. Psychological factors such as motivation, ability, and interests can also play a role in learning [5,6].

Among studied psychological factors, metacognition, or the ability to think about thinking, was found to be rather important to learning and learning adaptation. Metacognition in brief is awareness and control of thinking for learning which provides a way to understand cognitive monitoring [7]. Learners with metacognitive skills are more self-aware as critical thinkers and problem solvers, enabling them to actively approach knowledge gaps and problems. Because of the function of self-monitoring, students can be more aware of the state of their study process and problems in learning. They later can consciously adjust their tactics to deal with encountered problems in learning. Therefore, metacognition is beneficial to learning adaptation [8,9].

Certainly, there are individual differences in the possession of metacognition and applying metacognition in learning [10]. Personality as a stable indicator of individual differences influences metacognition [11] as well as learning adaptation [12]. A recent meta-analysis (*N* = 15,609) found the General Factor of Personality (GFP) from the Big Five personality traits which highly indicates ego-resiliency (*r* = 0.93) – an overall ability to adaptively modify the level of self-control to match the circumstances [13].

Compared with the concept of ego-resiliency, metacognition appears to convey a similar meaning but specifically refers to learning adaptation. In other words, metacognition appears as a specific instance of applying ego-resiliency in the context of learning adaptation. Therefore, this study proposes to use the GFP, which indicates ego-resiliency, to predict learning adaptation, serving as an analog to metacognition. By considering the GFP, this study offers a novel perspective for understanding how a comprehensive personality structure (GFP) influences learning adaptation, beyond the traditional focus on individual personality traits.

Next, this study will assess the extent of overlap between the GFP and metacognition. This assessment could statistically test whether ego-resiliency and metacognition are indeed analogs. Following this, the study will compare the unique influences of the GFP and metacognition on learning adaptation. Furthermore, it will test how the GFP and metacognition sequentially impact learning adaptation. This latter test will help answer the question: Which is the root cause of learning adaptation, the GFP or metacognition? If the GFP is found to have a similar or higher correlation with learning adaptation than metacognition, it could potentially replace the latter in predicting student learning adaptation. Practically speaking, personality measures are more often utilized than metacognition measures in enrollment procedures. This could streamline assessment procedures by reducing the number of required measurements and, consequently, the time invested in such measurements.

### 1.1 Metacognition

Ever since Flavell [7] introduced the notion of metacognition to understand the influence of cognitive monitoring, many researchers have paid much attention to unveiling its functioning in learning [14,15]. A common knowledge today is that metacognition plays a rather important role in students’ learning. As Wang et al. [16] summarized, students with strong metacognitive skills tend to learn more and perform better than those who are still developing their metacognition. Metacognition enables students and other learners to monitor, plan, and control their mental processes. Moreover, it enables learners to better assess the depth of their knowledge in a domain and allows learners to transfer/apply their knowledge and skills to new situations/domains and choose more effective learning strategies. Given those different functions, metacognition was speculated to have different dimensions.

Flavell [7] proposed that metacognition consists of both metacognitive knowledge and metacognitive experiences or regulation. Metacognitive knowledge refers to acquired knowledge about cognitive processes and knowledge that can be used to control cognitive processes. Moreover, Flavell described metacognitive experiences as *“any conscious cognitive or affective experience that accompanies or pertains to any intellectual enterprise”*. He believed that metacognitive experiences are more likely to occur during a task due to the importance of individuals’ decisions and actions in the endeavor.

Later researchers additionally considered metacognitive skills which refer to the skills that allow individuals to monitor and control their cognitive processes. These skills include planning, monitoring, evaluating, and regulating one's learning [17]. Stanton et al. [10] recently refined the hierarchical structure of metacognition. They claimed that metacognition included two main dimensions: metacognitive knowledge and metacognitive regulation.

By summarizing the existing classifications of metacognitive components in the literature, it can be speculated that metacognitive knowledge provides fundamental underpinnings for metacognitive regulation while metacognitive skills refer to effectively applying metacognitive knowledge in learning. During the procedure of metacognitive activity, learners perceived metacognitive experiences.

### 1.2 Personality and learning

Personality is commonly acknowledged to persistently produce a widespread influence on individuals' life, work, and learning [12]. In education and students' learning, personality plays a vital role. In past decades, educational psychologists commonly used the Big Five personality traits to study individual differences in learning-related activities [18,19]. The Big Five traits are often referred to as openness to experience, conscientiousness, extraversion, agreeableness, and neuroticism. Openness to experience includes aspects such as intellectual curiosity and creative imagination. Conscientiousness refers to organization, productiveness, and responsibility. Extraversion is characterized by sociability and assertiveness. Agreeableness includes compassion, respectfulness, and trust in others. Neuroticism refers to tendencies toward anxiety and depression [12,20]. Numerous studies tested the influence of those five traits on individuals' decision-making in educational activities. For example, the Big Five traits were found to be associated with individuals' choice of majors [18]. Mammadov [19] identified conscientiousness to be the most important trait in the Big Five for individuals’ academic performance. Boekaerts [21] extensively discussed the influence of personality on the learning process, learning behaviors, and learning experiences.

Nevertheless, to study learning adaptation, comprehensive personality characteristics with adaptive meaning seem to be more suitable, compared to specific personality traits (such as the Big Five) since learning adaptation is a complex procedure that needs many personality traits to work together in a way that individuals can effectively adapt to the learning environment. Thus, ego-resiliency was selected in the study as it indicates a cluster of characteristics for adaptively modifying the level of self-control to match the circumstances [22]. Learning adaptation appears to be an application of ego-resiliency, where individuals adaptively modify their level of self-control to meet the demands of the learning environment. Li et al. [23] recently indeed found the influence of ego-resiliency on school adaptation among primary school lower-grade children.

### 1.3 The general factor of personality

A recent study [13] found that ego-resiliency can be almost fully indicated by a General Factor of Personality (GFP) of the Big Five personality traits (*r* = 0.93). That finding suggested that the GFP presents a way of reorganizing personality traits in such a way that individuals can better fit the environment [24]. The GFP is statistically a lion-share of intercorrelations of the Big Five traits [25,26]. Not only in the Big Five traits but also in almost all known and less-known personality measurements, a GFP can be found [[27], [28], [29], [30], [31], [32]] with of course a few exceptions [33,34]. For its theoretical meaning, substantial evidence supported that the GFP indicated a cluster of social characteristics in personality that enabled individuals to adapt well to the social environment. As a result of such a good adaptation, individuals with high GFP scores are more able to approach their social goals [35]. For example, the GFP was found to positively link with likeability and popularity and the winning of student leadership positions in the classroom [36,37], leadership-efficiency and -emergence [38,39], and employees' job performance [40,41] in the workplace, better neighborhood, and higher salary regarding individuals’ life condition [42,43]. Excluding those examples in the industrial civilization, Van der Linden et al. [44] even found that in a less-industrialized society in the Amazon area, high GFP males tended to have more numbers of (living) offspring.

Like any rising notion in social science, the notion of GFP was also criticized as some thought it to be an artifact representing a measurement error [33,45]. To respond to the criticisms Dunkel et al. [46] conducted a study in which they compared measures of socially desirable response bias and positive self-evaluation and a rater-based measure of social effectiveness in explaining the variance of self-reported GFP. As found, although each of those three measures significantly explained the variance of self-reported GFP, rater-based social effectiveness often explained the largest part of the variance. Based on this finding, the authors suggested that self-reported GFP may contain three interrelated facets but social effectiveness is its most important ingredient. Using a way of reverse inference, Pelt et al. [40] and Wu et al. [37] found that controlling for the GFP (as criticisms of the GFP suggested) rendered the Big Five to lose most significant correlations with criterion-related variables (leadership and leadership emergence respectively). Some Big Five traits even changed the direction of correlations (from + to -) once the GFP was controlled for. These findings suggest that the GFP is a root reason for the found correlation between the Big Five traits (branch) and leadership variables and controlling for the GFP (root) makes the Big Five lose most of their power in the prediction of leadership variables. For more extensive discussion about the debate of substantive and artifact explanations of the GFP, one can refer to previous articles [47,48]. As for the scope of the present study, the GFP as ego-resiliency would be of interest.

### 1.4 Current study

The study aims to test four hypotheses as follows.

Hypothesis 1 The GFP is linked to learning adaptation.

Hypothesis 2 The GFP overlaps with metacognition.

Hypothesis 3 The GFP is more strongly linked to metacognitive skills than to metacognitive knowledge and experiences.

Hypothesis 4 The GFP contributes more significantly to learning adaptation than metacognition does.

Hypothesis 1 tests whether the GFP is useful in predicting learning adaptation, while Hypothesis 2 examines the extent to which the GFP plays a similar role as metacognition. The formulation of Hypothesis 3 is based on speculations from previous studies on the GFP, suggesting that the GFP's power lies primarily in applying individuals' abilities (especially social abilities) to optimize their fit with the social context [24]. Evidence supporting this can be found in Van der Linden et al. [49], where the GFP was found to be more strongly linked to trait emotional intelligence (the tendency/readiness to apply emotional intelligence) than to ability emotional intelligence (possession of emotional intelligence). A similar finding was recently reported by Wu et al. [50] in a preprint manuscript, who found that the GFP is more strongly linked to Creative Self-beliefs (the confidence in having/applying creativity) than to creativity (possession of creativity). The final Hypothesis 4 aims to compare the GFP with metacognition in terms of their impact on learning adaptability. If the GFP shows a higher correlation with learning adaptation than metacognition, it could potentially replace metacognition in predicting learning adaptation among students.

## 2 Method

### 2.1 Re-analysis

To test four hypotheses of interest, the present study re-analyzed the outputs from a psychological study published in a Chinese scientific journal. For testing correlations among adolescents’ Big Five personality traits, metacognition, and learning adaptation, Nie et al. [51] collected data from four middle/high schools. The sections below elaborate on the participants and measurements in the original study. Table 1 displayed reported correlations among tested variables from the original study of Nie et al. Note that when the authors published the study, no ethical approval was required.

**Table 1:** Correlations among the Big Five personality traits, the GFP and metacognition (sum scores and dimensions).

Variables	1	2	3	4	5	6	7	8	9	10
1.O	1.00									
2.C	0.11^b^	1.00								
3.E	0.05	0.36^b^	1.00							
4.A	0.03	0.33^b^	0.34^b^	1.00						
5.N	−0.06	−0.44^b^	−0.36^b^	−0.37^b^	1.00					
6.Skills	0.10^b^	0.40^b^	0.21^b^	0.24^b^	−0.45^b^	1.00				
7.Knowledge	0.07^a^	0.32^b^	0.13^b^	0.11^b^	−0.12^b^	0.29^b^	1.00			
8.Experience	0.11^b^	0.31^b^	0.17^b^	0.17^b^	−0.40^b^	0.58^b^	0.22^b^	1.00		
9.Metacognition	0.13^b^	0.48^b^	0.23^b^	0.24^b^	−0.45^b^	0.88^b^	0.62^b^	0.76^b^	1.00	
10.Learning adaptation	0.13^b^	0.52^b^	0.32^b^	0.26^b^	−0.32^b^	0.42^b^	0.22^b^	0.29^b^	0.43^b^	1.00
	Factor loadings of the GFP					Correlations with the GFP				
11.GFP	0.14	0.74	0.56	0.50	−0.60	0.57^b^	0.29^b^	0.47^b^	0.67^b^	0.61^b^

*Note.* Knowledge, metacognitive knowledge; Skills, metacognitive skills; Experience, metacognitive experience.

**Fig. 1:**
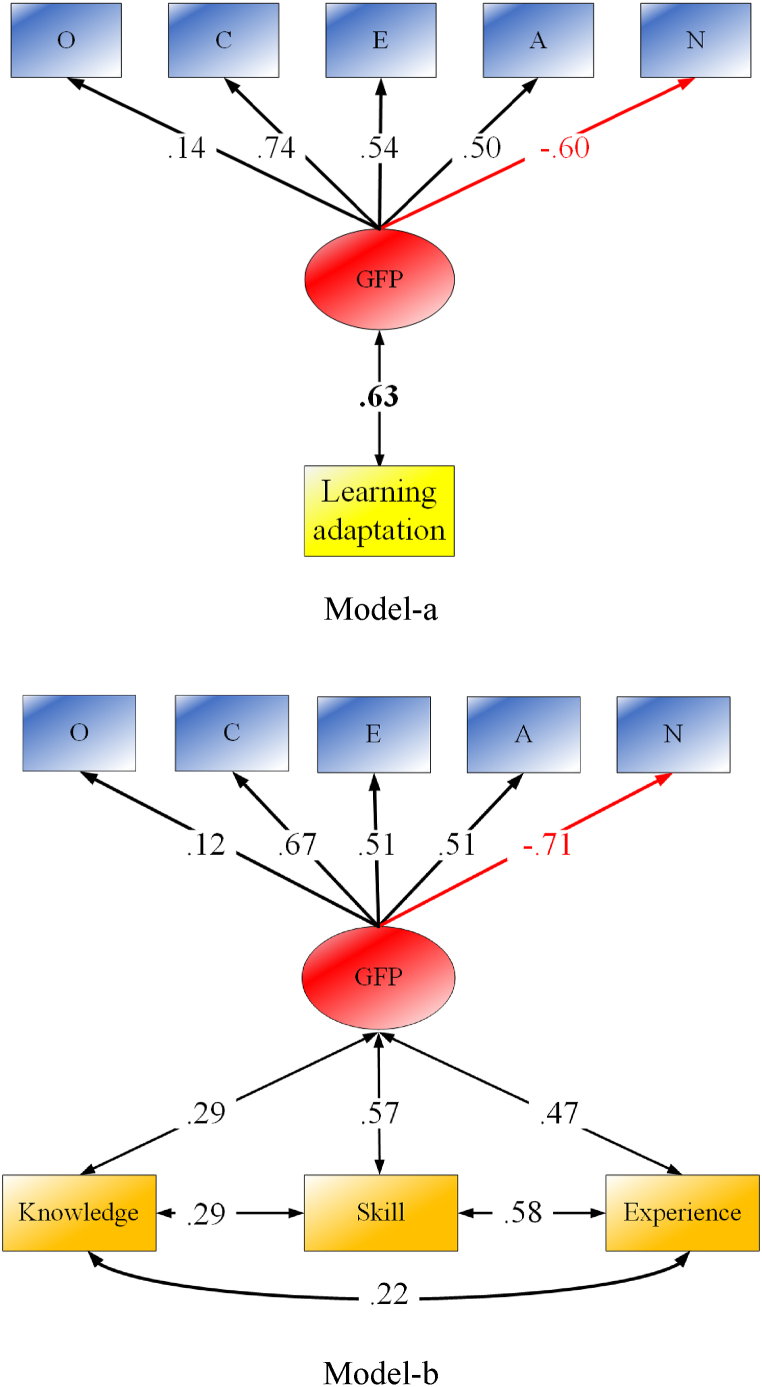
Correlations between the GFP and learning adaptation (Model-a) and between the GFP and dimensions of metacognition (Model-b). Note. Knowledge, metacognitive knowledge; Skills, metacognitive skills; Experience, metacognitive experience.

Factor loadings were extracted from the CFA model (Model-a) in Fig. 1. Correlations of the GFP with variables were extracted from SEMs in Model-b in Fig. 1 and from Model-a in Fig. 3.

a indicates *P* < .05.

b indicates *P* < .01.

### 2.2 Participants

Nie et al. randomly sampled 1050 students from four middle/high schools in Guangzhou city and asked those students to fill out three questionnaires (details can be seen in the section below). At length, 985 valid questionnaires were obtained with a validity rate of 93.81 %. The sample consisted of 452 male students and 533 female students with 314 first-year middle school students (about 12–13 years old), 218 s-middle school students (about 13–14 years old), 213 first-year high school students (about 15–16 years old), and 249 s-year high school students (about 16–17 years old). Due to the data collection period being close to the High School Entrance Examination (Zhongkao) and National Entrance Examination (Gaokao), the third-year middle and high school students (about 14–15 and 17–18 years old, respectively) were extremely busy preparing for their exams and had no time to participate in the study.

### 2.3 Measurements

#### 2.3.1 Learning adaptive behavior

The Adolescent Social Adaptive Behavior Scale [51] was used to assess learning motivation, learning habits, learning methods, learning satisfaction, and the use of learning resources. Each item includes several sub-items. For example, under “learning motivation”, there are 6 sub-items such as “striving to improve one's own quality”. For each sub-item, the answer is “yes” or “no”, scored as “1 point” and “0 point” respectively. The item score is the sum of the scores of each sub-item. The higher the score, the better the subject's good learning adaptive behavior ability or consciousness. In that study, the alpha coefficient of this scale was 0.68.

#### 2.3.2 The Big Five personality brief scale (NEO-FFI)

The Chinese version of NEO-FFI [52] was used to assess neuroticism, conscientiousness, extraversion, agreeableness, and openness. The scale consists of 60 items with 5 levels from “strongly disagree” to “strongly agree”. In this study, the alpha coefficients for neuroticism, conscientiousness, extraversion, agreeableness, and openness were 0.75, 0.76, 0.68, 0.65, and 0.63 respectively.

#### 2.3.3 Metacognition

The Metacognition Questionnaire [53] was used to assess metacognitive knowledge, metacognitive experience, and metacognitive skills. The questionnaire consists of three sub-questionnaires with a total of 26 items using a Likert-type 5-point scoring system. The higher the score, the stronger the metacognitive ability. In this study, the overall alpha coefficient was 0.81 and the alpha coefficients for each factor were 0.71, 0.70, and 0.72 respectively.

## 3 Results

### 3.1 Confirmatory Factor Analysis

Confirmatory Factor Analysis (CFA) was used to extract the GFP from the Big Five traits. The model displayed a perfect model fit (*χ*^*2*^ = 8.62, *df* = 5, CFI = 0.99, TLI = 0.99, RMSEA = 0.03). Factor loadings were 11, 64, 56, 55, and −0.67 for openness, conscientiousness, extraversion, agreeableness, and neuroticism respectively. Although openness contributes to the general factor much less than the other four traits, it still needs to remain in the CFA model for the completeness of the GFP (Van der Linden et al., 2010).

### 3.2 Correlations of the GFP with learning adaptation and metacognition

**Fig. 2:**
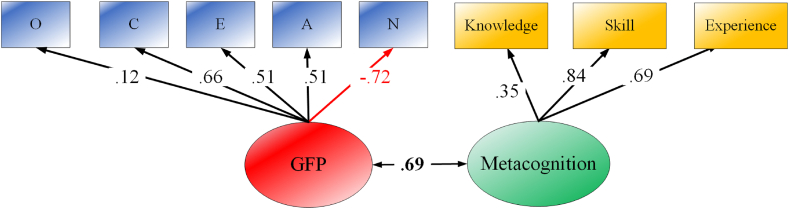
Correlation between the GFP and metacognition factor. Note. Knowledge, metacognitive knowledge; Skills, metacognitive skills; Experience, metacognitive experience.

The later structural equation models (SEM) test the correlation of the GFP with learning adaptation (Hypothesis 1). Model-a in Fig. 2 visualizes the model. As found, the GFP displays a high correlation with learning adaptation (*r* = 0.63; *χ*^*2*^ = 58.94, *df* = 9, CFI = 0.95, TLI = 0.92, RMSEA = 0.08).

To test **Hypotheses 2** and **3**, a series of SEMs were built. As found first in Model-b in Fig. 1, all three dimensions of metacognition significantly correlated with the GFP with metacognitive skills leading to the correlation (*r* = 0.57; *χ*^*2*^ = 112.76, *df* = 17, CFI = 0.94, TLI = 0.90, RMSEA = 0.08). Specific values of correlations can be seen in the lower bottom of Table 1. The difference test on those correlations confirmed that metacognitive skills have a significantly higher correlation with the GFP than metacognitive experiences (*Δr* = 0.10, *Z* = 3.05, *P* < 0.001) and knowledge (*Δr* = 0.28, *Z* = 7.73, *P* < 0.001). Following that, the next SEM was considered to test the correlation of the GFP with an overall metacognition factor which was extracted from its three dimensions. That model (see in Fig. 2) with a good model fit (*χ*^*2*^ = 116.49, *df* = 19, CFI = 0.94, TLI = 0.91, RMSEA = 0.07) displayed an overlap between the GFP and an overall metacognition factor (*r* = 0.69).

### 3.3 Relationship among the GFP, metacognition, and learning adaptation

**Fig. 3:**
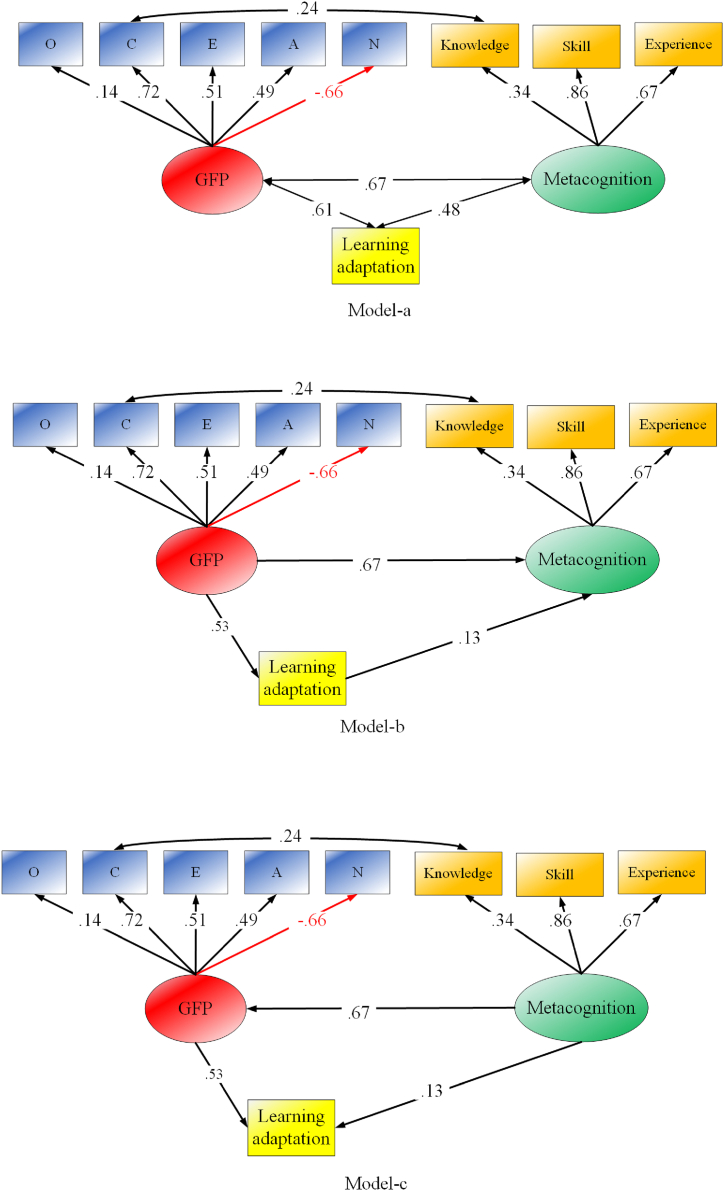
Relationship among the GFP, metacognition factor, and learning adaptation. Note. Knowledge, metacognitive knowledge; Skills, metacognitive skills; Experience, metacognitive experience.

To test Hypothesis 4, the next SEM first examined the correlations among the GFP, metacognition, and learning adaptation. As visualized in model-a of Fig. 3, the GFP was found to be highly correlated with either learning adaptation or metacognition while the latter two were found to correlate at a moderate level according to Cohen [54]. Specific values can be seen at the bottom of Table 1. The GFP correlated with learning adaptation (*r* = 0.61) significantly higher than metacognition (*r* = 0.48; *Δr* = 0.13, *Z* = 4.12, *P* < 0.001), suggesting that the GFP is more likely to be the root reason for the outcome of learning adaptation. Therefore, in a latter SEM (model-b in Fig. 3), a mediative regression model was built in which learning adaptation regressed directly on the GFP and metacognition while metacognition mediated the indirect effect of the GFP on learning adaptation (*χ*^*2*^ = 140.31, *df* = 24, CFI = 0.94, TLI = 0.91, RMSEA = 0.07). As found, the GFP highly influences learning adaptation (*β* = 0.67) as well as metacognition (*β* = 0.53). In comparison, metacognition displays a small impact on learning adaptation (*β* = 0.13). In that model, the direct effects of GFP→learning adaptation is 0.53 while the indirect effects of GFP→metacognition→learning adaptation is 0.09 (0.67 × 0.13). The direct effect occupied 85 % of total effects (0.62), and for the indirect effect, it is merely 15 %. To ensure the subjective selection of metacognition as a mediator is not the reason for the findings, the present study also tested the model in which the GFP is treated as a mediator while metacognition is an independent variable. Such a model (model-c in Fig. 3) displays an identical model fit as the metacognition-mediator model (model-b in Fig. 3) but a shrunk total effect (0.48). Meanwhile, the indirect effects of metacognition→GFP→learning adaptation (0.36) occupied 75 % of total effects. The direct effects of metacognition→ learning adaptation (0.13) contributed merely 25 % of the total effects. From both models, it can be reasoned that the GFP is the root reason for the outcomes of learning adaptation.

### 3.4 Incremental effects of the GFP over the Big Five

**Fig. 4:**
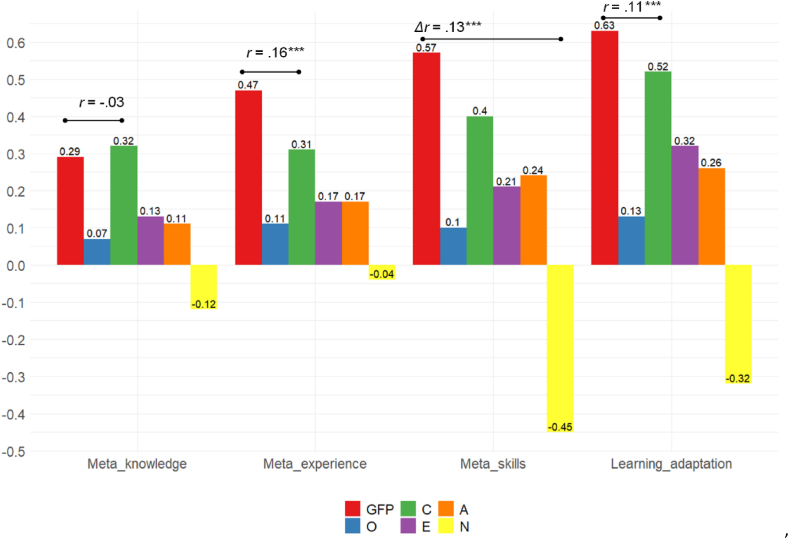
Comparison between the GFP and the Big Five traits in correlations with metacognition dimensions and learning adaptation.

To check to which extent the use of the GFP strengthens the prediction of learning adaptation in the Big Five context, the present study compared the GFP with the trait that leads to the correlations with either metacognition dimensions or learning adaptation among the Big Five. Fig. 4 visualizes the results. As can be seen, the GFP correlated with metacognitive skills and experience and learning adaptation significantly much higher than any Big Five trait. The only exception is metacognitive knowledge, with which, the GFP and conscientiousness shared a similar correlation (*Δr* = −0.03, Z = 0.73, *P* = 0.46).

## 4 Discussion

As metacognition and ego-resiliency similarly represent an adaptive adjustment to fit the requirement of the environment theoretically, the present study considered testing whether ego-resiliency can play the same role as metacognition in influencing learning adaptation. While the literature showed that ego-resiliency was almost fully presented by the General Factor of Personality/GFP extracted from the Big Five traits (Dunkel et al., 2021), the present study tested the GFP's influence on learning adaptation in comparison with metacognition.

The result first confirmed Hypothesis 1: a large influence of the GFP on learning adaptation supported the notion that the GFP is indicative of ego-resiliency – an ability to adaptively adjust self-control to fit well the environment. In the studied case, good adaptation in learning to approach good academic achievement is of course the most important requirement of the learning context, especially for Chinese middle school students who live in an atmosphere of exam-oriented education. Better academic achievements mean more chances to enter better universities, which subsequently largely decide students’ development in future careers [55,56].

Next, the GFP was found to significantly correlate with all dimensions of metacognition (Hypothesis 2) while it correlated with metacognitive skills rather higher than metacognitive experience and metacognitive knowledge (Hypothesis 3). That implied that the GFP is more associated with the application of metacognition rather than the possession of metacognition knowledge. This finding aligns with previous studies that underscored the relevance of the GFP in the application of social characteristics in real life such as the link of the GFP with likeability and popularity and winning student leader positions in classrooms [36,37], leadership efficiency and emergence [38,39], and employee job performance [40,41] in the workplace.

In addition, Wu et al. [50; preprint] recently meta-analytically found that the GFP significantly correlated with creativity (*ρ* = 0.22; *N* = 56748) but highly correlated with Creative Self-beliefs (*ρ* = 0.50; *N* = 19861) suggesting that the most relevance of the GFP relies on its applied characteristics. A high score on the GFP implied high motivation and confidence in applying creativity. Taken together, the GFP as ego-resiliency represents a readiness/tendency to adjust self-control and behavior to adapt to the environment.

Moreover, an almost high correlation between the GFP and metacognitive experience (*r* = 0.47) shed light on understanding the process of metacognition. Past knowledge emphasized the importance of consciously applying metacognition in learning. Along with that, Stanton et al. [10] provided many strategies for fostering metacognition to support student learning and performance. That is of course contributable to the effective application of metacognition in education. Meanwhile, the authors also issued a question that for different students, the suggested strategies work differently. A student's attitude or mindset would largely decide the effects of the application of metacognition in students' learning. For example, when starting to use a strategy for applying metacognition, a student might expect that strategy would not work while another student might believe in the effects of that strategy. Naturally, those two students later would report different effects of applying the same strategy. This example characterized how individual differences can largely influence the outcome of applying metacognition in learning. The present study contributes to specifying that individual difference. As the GFP was found to be rather stable (about 50 % of it is heritable) [57], its correlation with metacognitive experiences implied that parts of metacognition may be a stable characteristic akin to personality traits. The high GFP student would have more metacognitive experience than the low GFP student. Using the example given by Stanton et al. [10], high GFP students tend to more often use metacognitive strategies (skills) in their learning. Taking metacognitive-knowledges, -skills, and -experience altogether into account, the present study also tested overall metacognition in correlation with the GFP. A remarkable overlap was found.

Thereafter, the GFP and metacognition were compared in their correlations with learning adaptation. As found, the GFP (*β* = 0.53) displayed a much larger influence on learning adaptation than metacognition (*β* = 0.13). In a mediation SEM model, the GFP contributed most (85 %) effects on the influence to learning adaptation while metacognition had merely a small effect (15 %). By a reverse test in which the GFP was treated as a mediator while metacognition was an independent variable, the majority of the influence of metacognition on learning adaptation was found to go through the GFP. All these findings suggested that the GFP is the root reason for the outcome of learning adaptation.

Considering effect sizes, metacognition could be seemingly ignored when the GFP was included in predicting learning adaptation. This finding supports the notion of the GFP as ego-resiliency. Ego-resiliency, in definition, represents a general and fundamental mechanism of adaptive adjustment to fit the environment [58] while metacognition seems to be a specified ego-resiliency in the learning adaptation. Therefore, ego-resiliency can be considered a root of metacognition. The use of the GFP, namely ego-resiliency, seemed to be able to decrease the number of considered variables (say metacognition in this study) in predicting learning adaptation which is helpful to simplify the procedure of prediction in practice. Even so, that does not mean that metacognition is no longer useful in studying learning activities. As the present study only focused on learning adaptation, the utility of the GFP over metacognition can only be applied to learning adaptation. There are many other learning activities such as major learning, effective learning, and preparation for exams to which metacognition was found to be vital [6,8]. Moreover, for the issue of learning adaptation, metacognition also provides learning-directed knowledge to understand that activity while the GFP provides a more general explanation. In detail, studying metacognition enables us to find strategies that help students learn more effectively while the GFP helps explain to which extent those strategies work.

### 4.1 Relationship between GFP and metacognition

It is necessary to pay attention, nevertheless, that the relationship between the GFP and metacognition is complex and multifaceted. Both GFP and metacognition are related to self-awareness and self-regulation, and they can influence each other in a bidirectional manner [59]. One theoretical perspective is that GFP represents a higher-order personality trait that encompasses a range of self-regulatory abilities, including metacognition. In other words, GFP may provide a general framework for self-regulation, which can then be used to develop more specific skills, such as metacognition. Another perspective is that GFP and metacognition are both related to the development of self-awareness and self-regulation. Self-awareness is the ability to understand one's own thoughts, feelings, and motivations [60]. Self-regulation is the ability to control one's thoughts, feelings, and behaviors in order to achieve goals [61]. Both GFP and metacognition are necessary for effective self-awareness and self-regulation. The relationship between GFP and metacognition is likely to be bidirectional. GFP can influence metacognition by providing a general framework for self-regulation, while metacognition can influence GFP by helping individuals develop more effective strategies for adapting to their environment.

## 5 Limitations and future research

### 5.1 Common method variance

One limitation of the present study is that all measures were self-reported, which raises concerns about common method variance [62]. Common method variance occurs when the same method is used to measure multiple variables, which can inflate the correlations between those variables. To address this concern, future studies could use multiple methods to measure the constructs of interest. For example, GFP could be measured using self-report, peer-report, and behavioral observation. Metacognition could be measured using self-reporting, interviews, and performance on metacognitive tasks.

### 5.2 Generalizability

Another limitation of the present study is that the sample consisted of Chinese middle school students. It is unclear whether the findings would generalize to other populations, such as students from other countries or adults. Future studies could replicate the present study in different populations to examine the generalizability of the findings.

In addition to the suggestions above, future research could also explore the following questions: What is the relationship between GFP and metacognition in different cultures? How does GFP influence the development of metacognition? What are the long-term effects of GFP on learning adaptation?

## 6 Contributions and implications for practice

The present study found that in self-reported measures, the GFP, extracted from the Big Five personality traits a) overlaps with metacognition; b) impacts learning adaptation much more than metacognition; and c) a large part of the impact of metacognition on learning adaptation is mediated by the GFP. These findings together implied that when self-reporting measurement is used, the GFP is a better predictor of middle school students' learning adaptation than metacognition. In practice, given that the GFP can be extracted from the Big Five personality traits and the latter are commonly applied in middle/high school and college students' enrollment and evaluation, the use of the GFP to predict those students’ learning adaptation could reduce the number of required measurements and, consequently, the time invested in such measurements. Relevantly, educators could use measures of GFP to identify students who are at risk for poor learning adaptation. These students could then be provided with interventions to help them develop their metacognitive skills. Additionally, educators could use the findings of the present study to develop more effective teaching strategies. For example, they could use strategies that are designed to promote self-awareness and self-regulation (cores of the GFP and metacognition).

## Data availability statement

This is a reanalysis study using the outputs of a published study. The original data is not available but the reported correlation matrix and coding in R are accessible via the provided link: https://osf.io/ue83r/?view_only=cc7aec7673824fa1ae42dedabe15c276. Data were re-analyzed using R-studio, version 2023.03.0 with packages psych, ggplot2, dplyr, car, pwr, reshape2, semPlot, and corrplot. This study's design and its analysis were not pre-registered.

## Ethics approval

Ethical approval was not required for the present article as it involved the re-analysis of results from a published study. Review and/or approval by an ethics committee was not needed for that published study because when it was published, no ethical approval was required.

## CRediT authorship contribution statement

**Peiqian Wu:** Writing – review & editing, Writing – original draft, Visualization, Validation, Software, Project administration, Methodology, Investigation, Formal analysis, Conceptualization.

## Declaration of competing interest

The authors declare that they have no known competing financial interests or personal relationships that could have appeared to influence the work reported in this paper.
