# Supplementary material for: Beyond metacognition: The dominant role of the general factor of personality in learning adaptation
Source: Heliyon. 2024 Jul 25;10(15):e35147. doi: 10.1016/j.heliyon.2024.e35147 (PMC11328064; doi:10.1016/j.heliyon.2024.e35147)
Supplement: Multimedia component 1 [file mmc1.pdf]

这一部分有**两种类型**的题目，一种题目中的各种情况是按由难到易的顺序排列，只要求你把你所能做到的***最高水平的行为***选出来，在该选项后的数字上画圈（例如第1题，如果在家吃饭时，自己会炒简单的几个菜就餐，而不会根据需要独立的煮饭炒菜的话，请在“3”上画圈，并在方框里填上“3”）。另外一种题目要求你阅读***所有的***句子并用***“是”或“否”***作答（例如第2题）。

注：如果你没有机会做题目中所提及的事情，就按照如果给你机会的话，你所能完成的情况回答。

1．饮食就餐＊＊

1）在家需吃饭时

可以根据需要独立的煮饭炒菜	4	
自己会炒简单的几个菜	3	
能够使用微波炉加热饭菜	2	
会做快餐面	1	
从不自己做饭菜	0	□

2）在外面吃饭时

会在餐馆里点一席饭菜	4	
会叫买快餐如盒饭	3	
会在公共食堂买食物就餐	2	
会买包子、可乐等简单食物饮品	1	□
不会在外买食物	0	

2．卫生习惯	是	否	
每天洗澡	1	0	
身体有很强的异味如狐臭	0	1	
不定期更换内衣裤	0	1	
不自己清理指甲	0	1	
早晚固定刷牙	1	0	
时常忘记刷牙	1	0	
便后一般洗手	1	0	□
一般会餐前洗手	1	0	

3．照顾自己	是	否	
1）穿衣.			
知道在正式和非正式场合选择不同的服装	1	0	
如果没有他人的提醒，经常会穿不合适的衣服	0	1	
如没人提醒，会穿脏的或有污渍的衣服	0	1	
如没人提醒，会穿颜色搭配不协调的衣服	0	1	
如果没有他人帮助，会穿不合适的衣服	0	1	
如没人提醒，会穿破的或褶皱的衣服	0	1	
不知道在不同天气下穿雨衣、套鞋等特殊衣物	0	1	□

2）其他

中国知网 https://www.cnki.net

会照顾自己的身体（如更换湿衣服）	1	0	
处理轻伤（如割伤、烧伤）	1	0	
注意防范电源出口或插座的危险	1	0	
注意烫的食物、饮料、碗盘或锅存在的危险	1	0	
经常不注意可能存在的危险	1	0	□

4. 衣物护理 *	是	否	
独立使用家庭洗衣机、干衣机	1	0	
注意到衣服掉了钮扣或有小洞，或补衣服	1	0	
会熨平自己的衣服	1	0	
会手洗自己的衣服	1	0	
在需要时把鞋擦洗干净	1	0	
能够把衣服叠好放在衣橱或衣柜里	1	0	
能够把衣服挂起来而无需提醒	1	0	
从不参与做衣服洗熨的事	0	1	□

5. 方向感 *		
从单位、学校或家里走出几个街区后不迷失方向	3	
在单位院子里或离家几个街区的地方四处走动而不迷失方向	2	
独自在单位或家里的院子里四处走动	1	□
无论何时离开自己的生活范围都会迷路	0	

6. 利用交通工具	是	否	
会驾驶摩托车或小汽车(有驾照)	1	0	
会独自乘坐火车、长途公共汽车或飞机	1	0	
会独自乘坐出租车	1	0	
能够独自乘坐地铁或城市公共汽车走不熟悉的路线	1	0	
那个独自乘坐地铁或城市公共汽车走熟悉的路线	1	0	
会骑单车(自行车)外出上学	1	0	□

7. 外出活动	是	否	
能够独自参加组团旅游			
能去独自参加娱乐活动（如看电影等）并回家而无需照顾	1	0	
能去独立上学或回家而无需照顾	1	0	
能自己安全地过马路	1	0	□

8. 安全	是	否
知道可能存在的危险（如远离泳池的深水区、不搭坐陌生人的车、在车上系安全带等）	1	0
遵守红绿灯的指示	1	0
过马路时两边看并在必要时耐心等待	1	0
注意保管自己的财务	1	0

中国知网 https://www.cnki.net

不能认识到可能的危险	1	0	□

9. 打电话

	是	否	
会打国际长途电话	1	0	
会打国内长途电话	1	0	
会用磁卡收费电话	1	0	
会用固定电话打电话	1	0	
会用手机接打电话	1	0	
会用手机收发短信息	1	0	
知道报警电话和火警电话	1	0	□
知道医院救护电话	1	0	

10. 利用公共设施

	是	否	
知道邮资，从邮局购买邮票	1	0	
会到邮局寄领物品和汇款	1	0	
知道如何以及到哪里看医生	1	0	
知道去哪里购买必须学习和生活用品	1	0	
知道知道书店及怎样去	1	0	
知道图书馆并曾使用过	1	0	
知道社区附近的运动场地以及怎样去和使用	1	0	□

11. 劳动技能

	是	否	
会简单修理家电（如录音机、电视机遥控器）	1	0	
会简单修理自行车	1	0	
会换灯泡、电池	1	0	
会使用家用电器（如洗衣机、空调机、电视机等）	1	0	
会做家庭卫生工作，如拖地、倒垃圾等	1	0	
不能做任何工作	0	1	□

12. 钱的使用

很好地管理和支配自己的钱	4	
清楚收支状况，但不会使用银行设施	3	
购物会计算和找零	2	
会用钱，但不能正确了解收支状况	1	
不会用钱	0	□

13. 银行业务

	是	否	
独立使用银行设施	1	0	
在帮助下对帐户进行操作	1	0	
能填写存款和取款单	1	0	
有银行卡——会使用取款机	1	0	□

14. 预算

	是	否
为了某个特殊的目的存钱或积蓄	1	0
对日常开支进行预算	1	0
有计划地花钱	1	0

中国知网 https://www.cnki.net

控制自己的主要开支	1	0

15. 在附近购物

到几个商店去选购几种不同的东西	4
到一家商店去指明购买一种东西	3
不需要购物单去进行一些简单的购买	2
拿着购物单去进行一些简单的购买	1
不能被派去买东西	0

16 购物

独立买自己的衣物	5
买自己衣物上的配饰物	4
进行小宗购买（如糖、饮料）而无需帮助	3
在少许监控下购物	2
在严格陪伴监控下购物	1
不会购物	0

17. 学习态度	是	否
上学迟到而没有正当的理由	0	1
经常旷课	0	1
如不能经常得到监督或鼓励的话，就不能完成作业	0	1
我上课会走神或做小动作	0	1
上课不经许可就离开座位	0	1
时常抱怨学校或老师	0	1

18. 学习习惯	是	否
一般我会对功课进行预习	1	0
一般我会做完作业再去玩	1	0
我总是认真完成作业	1	0
我会对学习中的问题进行积极思考	1	0
遇到学习困难，我会向老师或他人请教	1	0
我喜欢对自己的学习进行总结	1	0

19. 学习方式	是	否
我有自己的学习计划	1	0
我的学习目标明确	1	0
我喜欢死记硬背知识	1	0
如果不理解，我记不住知识	1	0
我喜欢独立思考和完成学习任务	1	0
我愿意和同学一起学习和讨论问题	1	0
喜欢开放式的考试题	1	0

20. 利用学习资源	是	否

中国知网 https://www.cnki.net

会使用自己的学习工具书（如字典、参考资料）	1	0
会向老师、同学借学习资料	1	0
会到学校图书馆查资料或阅读	1	0
会到市区图书馆查资料或阅读	1	0
会使用互联网收集资料	1	0

21．学习动机	是	否
刻苦学习，报效国家和社会	1	0
为了提高自己的素质而努力学习	1	0
我总是希望成绩超过其他人	1	0
学习就是为了将来考个好大学、找个好工作	1	0
认真学习是为了报答父母和家庭	1	0
当我做对了一道难题时会觉得非常高兴	1	0

22．学习满意感	是	否
我的学习效率一般比较高	1	0
学习时很愉快	1	0
我很喜欢现在的学校	1	0
我对我现在的班级感到满意。	1	0
我对自己现在的成绩感到满意	1	0
我对现在的班主任和任课老师都很喜欢	1	0

23．时间	是	否
会用一般钟表看时间，而且能准确到分钟	1	0
会用电子钟表看时间	1	0
能看懂时间间隔（如3：30和4：30之间）	1	0
能理解对相同时间的不同表达，如九点四十五也可表述为十点差一刻	1	0
能把钟表上的时间与各种活动、事件联系起来	1	0

24．时间概念	是	否
知道中秋节和端午节的时间	1	0
知道我国的国庆节和建军节的时间	1	0
知道圣诞节的时间	1	0
知道教师节的时间	1	0
知道3.15的含义	1	0
知道元旦和春节的含义	1	0

B．语言发展

25．阅读与写作	是	否
会写演讲报告	1	0
能够用英语或其他外语写日记	1	0
会写信	1	0
能很好地完成申请表主要项目的填写	1	0

中国知网 https://www.cnki.net

注意利用读报理解时事政治和社会新闻	1	0	
为娱乐而看书、报纸或杂志	1	0	□

26. 对话

能够用英语或其他外语进行流利的交谈	4	
能够进行简单的英语或其他外语会话	3	
会讲流利的普通话	2	
会说用一些普通话交谈	1	
不会讲普通话	0	□

27. 社交语言 * *	是	否	
能够听懂别人的言外之意	1	0	
能够接受他人的批评	1	0	
可以劝说别人	1	0	
谈吐明智、幽默	1	0	
给别人讲关于运动、家庭和团体活动等事情	1	0	
使用“请”、“谢谢”等短语	1	0	□

28. 主动

自发开始进行自己的大多数活动，如学习、运动等	3	
主动询问是否有事情要做或探索周围环境，如家里、学校等	2	
只有在被分派任务或给予指令后才做事	1	
不参与被分派的活动，如做课室卫生等	0	□

29. 被动	是	否	
需要不断的鼓励才能完成任务	0	1	
必须被要求或指派才做事	0	1	
没有抱负	0	1	
似乎对做什么事情都没有兴趣	0	1	
因为浪费时间而最后才完成任务	0	1	
需要靠他人的帮助完成任务	0	1	□

30. 注意

注意可以集中注意于有目的的活动超过连续 1 天以上，如上课、做作业等	4	
可以集中注意于有目的的活动将近 4 时以上	3	
可以集中注意于有目的的活动将近 3 小时以上	2	
可以集中注意于有目的的活动将近 2 小时以上	1	□
不能集中注意于有目的的活动达到 1 小时	0	

31. 坚持	是	否
不能组织活动	0	1

中国知网 https://www.cnki.net

很容易气馁	0	1	
不能完成任务	0	1	
经常一件事没做完又去做另一件事	0	1	
需要不断鼓励才能完成任务	0	1	□

32. 休闲活动

组织较为复杂的休闲活动，如外出郊游、安排体育活动，或安排时间玩电脑游戏等	4	
对某种嗜好有强烈的兴趣，如收集邮票等	3	
能参与已经组织好的休闲活动	2	
参与简单的休闲活动，如看电视、听收音机等	1	
甚至连最简单的休闲活动都不会为自己安排	0	□

33. 个人财产

非常可靠，总能照顾好个人财产	3	
一般都很可靠，通常能照顾好个人财产	2	
不可靠，很少能照顾好个人财产	1	
一点都不负责任，从来不能照顾好个人财产	0	□

34. 一般责任

非常尽责并能假定很多责任——付出特别的努力，安排的活动通常都能完成	3	
通常都很可靠——努力完成职责，能保证被分派的活动完成	2	
不可靠——不努力完成职责，不能保证被分派的活动完成	1	
根本就不能完成职责	0	□

35. 个人责任	是	否	
通常都能保持自我控制	1	0	
知道守时	1	0	
能在知道下寻求并接受帮助	1	0	
如果遇到问题能向老师、家长等报告	1	0	□

36. 行为习惯

A. 规范

一般情况下，我会：	是	否	
公厕用后冲洗厕所	1	0	
遵守次序排队等候(如上车)	1	0	
有时，我会：			
随地吐痰	0	1	
随地大小便	0	1	
乱扔垃圾	0	1	
在公共场所大声喧哗	0	1	□

B. 合作

中国知网 https://www.cnki.net

主动向他人提供帮助	2
如果别人请求帮助的话，愿意帮助别人	1
从不帮助别人	0

☐

37. 为他人考虑	是	否
对别人的事情感兴趣	1	0
照顾别人的财物	1	0
在别人需要时，负责或管理别人的事务	1	0
考虑别人的感情	1	0

☐

38. 对他人的知觉 **	是	否
了解自己的家人	1	0
了解家人之外的其它人	1	0
知道别人的一些情况，诸如工作、地址、与自己的关系	1	0
知道与他/她关系密切的人的名字，如他/她的同班同学和邻居	1	0
知道不常碰到的人的名字	1	0

☐

39. 与他人相互影响	
在团体运动或活动中与他人相互影响	3
在至少较短的一段时间内与他人相互影响，如向别人展示或提供衣服、物品	2
与他人交往，但几乎没有相互影响	1
不以一种为社会所认可的方式对他人做出回应	0

☐

40. 参与集体活动	
发起集体活动（你是领导者或组织者）	3
自发地、积极地参与集体活动（你是积极的参与者）	2
在受到鼓励的情况下参与集体活动（你是被动的参与者）	1
不参加或退出集体活动	0

☐

41. 自私	是	否
拒绝轮流来负担	0	1
不和他人分享（经验、学习资料、信息）	0	1
如果不能按自己的意愿来做就会发脾气	0	1
打扰正在帮助别人的老师或同学	0	1

☐

42. 社交经验	是	否
容易与陌生人熟悉起来	1	0
不怕陌生人	1	0
力求结交朋友	1	0

中国知网 https://www.cnki.net

爱与所有人握手	1	0	
常跟着别人	1	0	□

43．使用计算机

自己可以安装电脑硬件	5	
会自己装卸电脑软件	4	
会用电脑编辑文档	3	
会利用计算机打字	2	
会在电脑上玩游戏	1	
不会使用电脑	0	□

44．网络技术

会制作网页	5	
会利用网络收集信息资料	4	
有自己的 QQ 号，会 QQ 室聊天	3	
会收发 E-mail	2	
知道怎样上网	1	
以上都不懂	0	□

45．上网

在使用互联网时，您主要进行下列哪些操作：	是	否	
用聊天软件与同学、朋友或陌生人聊天	0	1	
玩游戏或看电影	0	1	
浏览色情网站	0	1	
收发电子邮件	1	0	
浏览各种论坛	1	0	
查找有关学习资料	1	0	
看新闻	1	0	□

46．网络依恋

	是	否	
过去 7 天内平均上网每天超过 2 小时	1	0	
不上网时仍在想网络内容	1	0	
不能上网感到无聊和焦虑	1	0	
上网时间经常超出预期	1	0	
想不上网但无法自控	1	0	
因上网不能完成作业或逃学	1	0	
因上网与家长冲突	1	0	□

47．身体发育 *

	是	否	
身体健康	1	0	
身体各器官和机能正常	1	0	
身体发育正常	1	0	
身体存在某种器官（如听觉、肢体运动）缺陷	0	1	□

48．运动

中国知网 https://www.cnki.net

是校级以上运动员	3		
至少会二种以上运动项目，如乒乓球、游泳	2		
会一种器材运动项目，如打篮球、羽毛球	1		
除跑步外不会任何运动项目	0		□

49．异性交往

	是	否	
有性体验	1	0	
有恋爱经验	1	0	
能够与异性同学正常来往	1	0	
和异性交往感到紧张	0	1	□
没有与同龄异性交往	0	1	

50．性知识

	是	否	
能够控制的性冲动	1	0	
知道避孕方法	1	0	
懂得与异性交往的界线	1	0	
了解男女的身体结构和差异	1	0	□
没有多少性知识	1	0	

附加题（适用于女性）

无人帮助或提醒也可以在月经期照顾好自己	3	
在月经期可以较好地照顾自己	2	
在月经期会换卫生巾	1	□
上面所有这些事都没做	0	

（注：＊表示在初测之后被删除的题项，＊＊ 表示在正式测验之后经检验，项目负荷太低而被删除的题项 ）

附录 6： 青少年社会适应行为调查表（2）

指导语：

这一部分只有一种题型，要求你回答一些以下行为发生的频率。请根据自己的实际情况作出回答。

如从未发生（用 N 表示），请在“0”上画圈；

如偶尔发生（用 O 表示），请在“1”上画圈；

如经常发生（用 F 表示），请在“2”上画圈。

然后，请把每一题下各个句子的评分相加，把得数写在方框里。

另外，每题最后有一行空格，如果你还有该题中的句子所没有提及相关行为（只限一种），就请填写在空格上（必须是具体的例子）。

中国知网 https://www.cnki.net

这一部分共有37道题和1道附加题

	从未发生	偶尔	经常发生
1、威胁或施加身体暴力	N	O	F
使用威胁性的手势	0	1	2
间接给他人造成伤害	0	1	2
向别人吐唾沫	0	1	2
推、抓或掐被人	0	1	2
拉扯别人的头发、耳朵等	0	1	2
咬别人	0	1	2
踢、打或掌掴他人	0	1	2
向别人投掷东西	0	1	2
掐别人的脖子	0	1	2
用东西当武器打别人	0	1	2
伤害动物	0	1	2
其它________	0	1	2
2、粗暴或发脾气			
哭喊、尖叫	0	1	2
边摔东西边跺脚	0	1	2
跺脚、尖叫、大声喊叫	0	1	2
倒在地上，尖叫或大声喊叫	0	1	2
其它________	0	1	2
3. 取笑或讲他人闲话			
讲别人闲话	0	1	2
虚构或夸大别人的事	0	1	2
取笑别人	0	1	2
作弄别人	0	1	2
开别人的玩笑	0	1	2
其它________	0	1	2
4. 指挥或操纵他人	N	O	F
试图告诉别人该做什么	0	1	2
要求别人为自己提供服务	0	1	2
摆布别人	0	1	2
造成他人之间的打斗	0	1	2
操纵他人，使其陷入困境	0	1	2
其它________	0	1	2
5. 使用粗鲁的、威胁性的语言			
使用“傻瓜”、“脏货”等敌意的语言	0	1	2
诅咒、漫骂或说脏话	0	1	2
叫嚷着发出暴力威胁	0	1	2
口头威胁他人，暗示要施加身体暴力	0	1	2
其它________	0	1	2

中国知网 https://www.cnki.net

6. 对挫折的不良反应			
自己犯错却责备别人	0	1	2
受挫时退缩或撅着嘴板着脸	0	1	2
受挫时心烦意乱	0	1	2
其它________________	0	1	2
7. 扰乱其他人的活动			
总是碍别人的事	0	1	2
干涉别人的活动（如挡道等）	0	1	2
扰乱别人的工作或学习	0	1	2
搞乱别人正在玩的东西（如扑克等）	0	1	2
从别人手里抢夺东西	0	1	2
其它________________	0	1	2

	从未发生	偶尔	经常发生
8. 忽视规则或惯常的程序	N	O	F
对规则持消极态度但通常都会遵守	0	1	2
很不情愿但又不得不排队等候（如买票）	0	1	2
破坏规矩或规则（如不遵守交通信号）	0	1	2
拒绝参加要求参加的活动	0	1	2
其它____________________	0	1	2
9. 抵制以下指令、要求或命令			
如果别人直接命令你的话会觉得难过	0	1	2
假装没有听到或不执行指令	0	1	2
不注意听指令	0	1	2
拒绝做指定的工作	0	1	2
在做指定的工作之前要犹豫很久	0	1	2
做与要求相反的事	0	1	2
其它____________________	0	1	2
10. 对权威持无理的、反叛的态度	N	O	F
怨恨权威人士（如老师、领导等）	0	1	2
对权威人士不友好	0	1	2
嘲弄权威人士	0	1	2
说自己会炒权威人士的鱿鱼	0	1	2
说自己的亲友会来杀死或伤害权威人士	0	1	2
其它____________________	0	1	2
11. 缺席或迟到			
迟到要求到达的地方或活动	0	1	2
在离开后（如上厕所、出差等）没有返回应该返回的地方	0	1	2
没有经过允许就离开要求的活动场所	0	1	2
缺席例常活动（如上学等）	0	1	2
在家、宿舍或医院病房外待到很晚	0	1	2
其它____________________	0	1	2

中国知网 https://www.cnki.net

12. 逃离或试图逃离

试图逃离医院、家或学校	0	1	2	
逃离群体活动（如野炊等）	0	1	2	
逃离医院、家或学校	0	1	2	
其它＿＿＿＿＿＿＿＿	0	1	2	□

13. 在集体活动中行为失当

通过谈论无关话题打断集体讨论	0	1	2	
因拒绝遵守规则而扰乱游戏	0	1	2	
通过大声喧哗或捣乱而扰乱集体活动	0	1	2	
在上课、吃饭或开会时不坐下来	0	1	2	
其它＿＿＿＿＿＿＿＿	0	1	2	□

	从未发生	偶尔	经常发生	
14. 不尊重他人财产	N	O	F	
不归还借来的东西	0	1	2	
不经他人允许就使用其财产	0	1	2	
弄丢别人的东西	0	1	2	
毁坏别人的东西	0	1	2	
没有意识到自已和他人财产之间的区别	0	1	2	
其它＿＿＿＿＿＿＿＿	0	1	2	□
15. 不经允许就拿别人的东西				
被别人怀疑有偷窃行为	0	1	2	
将别人没有放好或锁起来的东西拿走	0	1	2	
从别人的口袋、钱包或抽屉拿东西	0	1	2	
通过开或撬锁拿别人的东西	0	1	2	
其它＿＿＿＿＿＿＿＿	0	1	2	□
16. 撒谎或骗人	N	O	F	
歪曲真相使之对自己有利	0	1	2	
在游戏、考试、作业等中作弊	0	1	2	
撒关于情景或形势的谎	0	1	2	
撒关于自己的谎	0	1	2	
撒关于别人的谎	0	1	2	
其它＿＿＿＿＿＿＿＿	0	1	2	□
17、损坏个人财产				
撕扯或咬自己的衣物	0	1	2	
弄脏自己的东西	0	1	2	
撕毁自己的书、杂志或其它东西	0	1	2	
其它＿＿＿＿＿＿＿＿	0	1	2	□
18. 损坏公共财产				
撕毁杂志、书或其它公共财产	0	1	2	
粗暴对待家具设施（踢、撞倒等）	0	1	2	
弄坏窗子	0	1	2	
用纸巾或其它固体堵塞厕所造成溢漏	0	1	2	□

中国知网 https://www.cnki.net

其它＿＿＿＿＿＿＿＿	0	1	2
19. 损坏他人财产			
撕扯或咬别人的衣物	0	1	2
弄脏别人的东西	0	1	2
撕毁别人的杂志、书等个人财产	0	1	2
其它＿＿＿＿＿＿＿＿	0	1	2

□

20. 有刻板行为	N	O	F
手指不停地击打作鼓声	0	1	2
脚不停地轻叩	0	1	2
手不停地动	0	1	2
不停地拍击、抓或擦自己	0	1	2
反复地摇动或晃动身体的某些部位	0	1	2
前后移动或滚动	0	1	2
前后摇摆身体	0	1	2
踱来踱去	0	1	2
其它＿＿＿＿＿＿＿＿	0	1	2

□

21. 人际交往方式不当			
说话时太靠近别人的脸	0	1	2
对着别人的脸吹气	0	1	2
对着别人打嗝	0	1	2
亲吻或舔别人	0	1	2
拥抱或挤压别人	0	1	2
不合适地抚摸别人	0	1	2
抓住别人不让走	0	1	2
其它＿＿＿＿＿＿＿＿	0	1	2

□

22. 有妨碍他人的口头或言语习惯	N	O	F
歇斯底里地哈哈大笑	0	1	2
大声说话或向别人叫喊	0	1	2
大声自言自语	0	1	2
不合适地笑	0	1	2
咆哮或发出嗡嗡声等让人不愉快的噪音	0	1	2
不断重复某个词或短语	0	1	2
学别人说话	0	1	2
其它＿＿＿＿＿＿＿＿	0	1	2

□

23. 有不受欢迎的口头习惯			
流口水	0	1	2
发出磨牙声	0	1	2
往地上吐痰	0	1	2
咬手指甲	0	1	2

中国知网 https://www.cnki.net

嚼或吮吸手指或身体的其它部位	0	1	2
嚼或耗损西衣物或其它不能吃的东西	0	1	2
吃不能吃的东西	0	1	2
坐在马桶上喝水或饮料	0	1	2
什么东西都往嘴里放	0	1	2
其它________________	0	1	2

24．有多动倾向

话过多	0	1	2
总是不愿意安静地坐一会儿	0	1	2
经常绕着房子或厅跑、跳	0	1	2
经常走动或坐立不安	0	1	2

25．有其它古怪的习惯或倾向	N	O	F
对睡觉或坐的地方过度挑剔	0	1	2
总是站在最喜欢的地方（如门边、窗边）	0	1	2
坐在震动的东西旁边	0	1	2
害怕上下楼梯	0	1	2
不想别人碰自己	0	1	2
别人触及自己时尖叫	0	1	2
其它________________	0	1	2

26．对自己施加身体暴力	N	O	F
咬或割伤自己	0	1	2
扇自己耳光或打自己	0	1	2
撞自己的头或身体的其它部位	0	1	2
扯自己的头发、耳朵等	0	1	2
抓或撕扯自己使自己受伤	0	1	2
把自己弄脏或在身上涂抹污物	0	1	2
故意惹别人打骂自己	0	1	2
弄痛他或她会有的痛处	0	1	2
戳自己耳朵、眼睛、鼻子或嘴里的东西	0	1	2
其它________________	0	1	2

27、有奇怪的或不受欢迎的习惯

什么东西都要闻一闻	0	1	2
在衣服口袋或鞋里放不合适的东西	0	1	2
从衣服里把线抽出来	0	1	2
玩弄穿的东西（如鞋带、钮扣）	0	1	2
收藏或穿戴奇特的东西（如安全别针、瓶盖等）	0	1	2
储藏东西，包括食物	0	1	2
玩唾液	0	1	2

中国知网 https://www.cnki.net

玩屎或尿	0	1	2
其它________________________	0	1	2

28. 不活跃	N	O	F
长时间坐或站在一个地方	0	1	2
什么也不干，只是坐着看别人	0	1	2
坐在椅子里睡着了	0	1	2
整天躺在地上	0	1	2
似乎对任何事都没有反应	0	1	2
其它________________________	0	1	2

29. 退缩	N	O	F
似乎不了解环境	0	1	2
很难接近或接触	0	1	2
在情感上反应迟钝或麻木不仁	0	1	2
翻白眼	0	1	2
表情呆滞	0	1	2
其它________________________	0	1	2

30. 害羞			
在社交场合胆小而害羞	0	1	2
在群体情景（如聚会）中埋着头	0	1	2
不能很好地与别人打成一片	0	1	2
喜欢独自一人	0	1	2
其它________________________	0	1	2

31. 有奇特的姿势或怪癖			
歪着头	0	1	2
坐着时用膝盖顶着下巴	0	1	2
用脚尖走路	0	1	2
两脚向上抬着躺在地上	0	1	2
走路时用手指塞住耳朵或用手抱着头	0	1	2
其它________________________	0	1	2

32. 往往会高估自己的能力	N	O	F
没有意识到自己的缺点或不足	0	1	2
对自己评价过高	0	1	2
谈论不现实的未来计划	0	1	2
其它________________________	0	1	2

33. 对批评的不当反应			
当被别人纠正错误时不说话	0	1	2
当被批评时走开或板着脸	0	1	2

中国知网 https://www.cnki.net

被别人纠正错误时大声叫喊	0	1	2
其它________________	0	1	2
34．要求过多的注意或表扬			
想要过多的表扬	0	1	2
妒忌给予别人的注意	0	1	2
要求过多的安慰	0	1	2
为得到注意而做出愚蠢的行为	0	1	2
其它________________	0	1	2
35．似乎觉得受到了迫害			
即时享受了平等权利时也抱怨不公	0	1	2
抱怨“没人爱我”	0	1	2
说“每个人都挑我的刺”	0	1	2
说“人们议论我”	0	1	2
说“人们都反对我”	0	1	2
怀疑他人	0	1	2
其它________________	0	1	2
36．有疑病倾向			
抱怨想象的（非真正有的）身体疾病	0	1	2
假装生病	0	1	2
病好后仍然作病态	0	1	2
其它________________	0	1	2
37．有其它情绪不稳定的迹象	N	O	F
没有明显的原因就产生情绪变化	0	1	2
抱怨老是做恶梦	0	1	2
在睡眠中惊叫	0	1	2
没有明确原因就哭	0	1	2
似乎不能控制情绪	0	1	2
难过时呕吐	0	1	2
在白天的活动中感觉不安全或害怕	0	1	2
谈论会引起不现实的恐惧的人或事	0	1	2
谈论自杀	0	1	2
**补充题**			
使用指定的（影响心理状态）的药物	N	O	F
使用镇静剂	0	1	2
使用止痛药	0	1	2
使用抗痉挛药	0	1	2
使用兴奋剂	0	1	2
其它________________	0	1	2

中国知网 https://www.cnki.net
