# Supplementary material for: Beyond metacognition: The dominant role of the general factor of personality in learning adaptation
Source: Heliyon. 2024 Jul 25;10(15):e35147. doi: 10.1016/j.heliyon.2024.e35147 (PMC11328064; doi:10.1016/j.heliyon.2024.e35147)
Supplement: Multimedia component 2 [file mmc2.docx]

**大五人格测验简版（NEO）**

**指导语：**以下是一些有关自我情感、态度和行为的陈述。请仔细阅读每个陈述，看看是否适合用来描述你自己。请逐题在相应的选项（1=非常不同意；2=不同意；3=没有意见；4=同意；5=非常同意）上划“√”，表明你同意该看法的程度。问卷不署名，敬请如实描述你的感受。回答无所谓对错好坏，不必花太多时间琢磨。

|  |  | 非常不同意 | 不同意 | 没有意见 | 同意 | 非常同意 |
| --- | --- | --- | --- | --- | --- | --- |
| 1 | 我不是一个喜欢担忧的人 | 1 | 2 | 3 | 4 | 5 |
| 2 | 我喜欢有许多人和我在一起 | 1 | 2 | 3 | 4 | 5 |
| 3 | 我很喜欢沉浸在在幻想和白日梦里，并探索所有可能性，任其蔓延和发展 | 1 | 2 | 3 | 4 | 5 |
| 4 | 我尽量对遇到的每一个人有礼貌 | 1 | 2 | 3 | 4 | 5 |
| 5 | 我把自己的东西收拾、保持得干净整洁 | 1 | 2 | 3 | 4 | 5 |
| 6 | 我有时感到痛苦和愤慨 | 1 | 2 | 3 | 4 | 5 |
| 7 | 我爱笑 | 1 | 2 | 3 | 4 | 5 |
| 8 | 我认为培养新的爱好很有趣 | 1 | 2 | 3 | 4 | 5 |
| 9 | 有时我会恐吓或哄骗别人，使他们干我想让他们干的事情 | 1 | 2 | 3 | 4 | 5 |
| 10 | 我能很好地安排时间，使各种事情按时完成 | 1 | 2 | 3 | 4 | 5 |
| 11 | 当我处于极度紧张状态时，有时觉得自己都要崩溃了 | 1 | 2 | 3 | 4 | 5 |
| 12 | 我喜欢那些可以让我单独完成而不会被别人打扰的工作 | 1 | 2 | 3 | 4 | 5 |
| 13 | 我对艺术和自然的表现形式着迷 | 1 | 2 | 3 | 4 | 5 |
| 14 | 有些人认为我自私和自我中心 | 1 | 2 | 3 | 4 | 5 |
| 15 | 我经常会遇到一些我没有充分准备的情况 | 1 | 2 | 3 | 4 | 5 |
| 16 | 我很少感到孤独或忧郁 | 1 | 2 | 3 | 4 | 5 |
| 17 | 我很喜欢和人们谈话 | 1 | 2 | 3 | 4 | 5 |
| 18 | 我认为让学生去听有争议的人的演讲只会让他们思想混乱，并把他们引向歧途 | 1 | 2 | 3 | 4 | 5 |
| 19 | 我是一个顽固、固执的人 | 1 | 2 | 3 | 4 | 5 |
| 20 | 我会尽心尽力去完成分配给我的所有任务 | 1 | 2 | 3 | 4 | 5 |
| 21 | 我常常会感到紧张和极度不安 | 1 | 2 | 3 | 4 | 5 |
| 22 | 我喜欢凑热闹 | 1 | 2 | 3 | 4 | 5 |
| 23 | 诗歌对我很少有影响甚至毫无影响 | 1 | 2 | 3 | 4 | 5 |
| 24 | 我知道我比多数人优秀 | 1 | 2 | 3 | 4 | 5 |
| 25 | 我的一系列目标很明确，并能逐步地实现它们 | 1 | 2 | 3 | 4 | 5 |
| 26 | 有时我感到自己毫无用处 | 1 | 2 | 3 | 4 | 5 |
| 27 | 我会避开人群 | 1 | 2 | 3 | 4 | 5 |
| 28 | 让思想无拘无束的漫游对我来说是件难事 | 1 | 2 | 3 | 4 | 5 |
| 29 | 如果我被人侮辱，我会尽量宽恕和忘却 | 1 | 2 | 3 | 4 | 5 |
| 30 | 通常我要花很多时间，才能安下心来工作 | 1 | 2 | 3 | 4 | 5 |
| 31 | 我很少感到恐惧或焦虑 | 1 | 2 | 3 | 4 | 5 |
| 32 | 我常常感到自己精力旺盛，活力十足 | 1 | 2 | 3 | 4 | 5 |
| 33 | 我很少注意到自己在不同环境中的心情或感受的变化 | 1 | 2 | 3 | 4 | 5 |
| 34 | 我倾向于把人往好里想 | 1 | 2 | 3 | 4 | 5 |
| 35 | 我工作勤奋，以便实现自己的目标 | 1 | 2 | 3 | 4 | 5 |
| 36 | 我常常为人们对待我的方式而生气 | 1 | 2 | 3 | 4 | 5 |
| 37 | 我是一个快乐而兴致勃勃的人 | 1 | 2 | 3 | 4 | 5 |
| 38 | 我体验到很多不同的感受和情绪 | 1 | 2 | 3 | 4 | 5 |
| 39 | 有些人认为我冷漠，只为自己打算 | 1 | 2 | 3 | 4 | 5 |
| 40 | 当我答应做一件事时，人们总相信我能坚持到底 | 1 | 2 | 3 | 4 | 5 |
| 41 | 当事情变得不顺利时，我常会感到泄气，并想放弃 | 1 | 2 | 3 | 4 | 5 |
| 42 | 与别人聊天并没让我得到更多的乐趣 | 1 | 2 | 3 | 4 | 5 |
| 43 | 读一首诗或观赏一件艺术品，有时会让我感到内心强烈的冲动 | 1 | 2 | 3 | 4 | 5 |
| 44 | 我的原则是讲求实际，不感情用事 | 1 | 2 | 3 | 4 | 5 |
| 45 | 我本应该是一个可靠和值得信赖的人，但有时做得却不是那么好 | 1 | 2 | 3 | 4 | 5 |
| 46 | 我很少悲伤或忧郁 | 1 | 2 | 3 | 4 | 5 |
| 47 | 我的生活是快节奏的 | 1 | 2 | 3 | 4 | 5 |
| 48 | 我没什么兴趣思索宇宙的本质或人类的现状 | 1 | 2 | 3 | 4 | 5 |
| 49 | 通常我会尽力考虑周全 | 1 | 2 | 3 | 4 | 5 |
| 50 | 我做事富有成效，总能及时、正确地完成任务 | 1 | 2 | 3 | 4 | 5 |
| 51 | 我常常感到无助，想要别人来解决我的问题 | 1 | 2 | 3 | 4 | 5 |
| 52 | 我是一个很活跃的人 | 1 | 2 | 3 | 4 | 5 |
| 53 | 我对动脑筋的事有很强的好奇心 | 1 | 2 | 3 | 4 | 5 |
| 54 | 如果我不喜欢某些人，我就让他们知道 | 1 | 2 | 3 | 4 | 5 |
| 55 | 我好像从来不能把事情做得井井有条 | 1 | 2 | 3 | 4 | 5 |
| 56 | 有时我感到非常羞愧，简直想躲起来 | 1 | 2 | 3 | 4 | 5 |
| 57 | 我宁愿独自行动，而不愿领着他人一起干 | 1 | 2 | 3 | 4 | 5 |
| 58 | 我经常对理论或抽象的概念感兴趣 | 1 | 2 | 3 | 4 | 5 |
| 59 | 在必要时，我会操纵、控制别人，以达到我的目的 | 1 | 2 | 3 | 4 | 5 |
| 60 | 我力求把所有事情都做得十全十美 | 1 | 2 | 3 | 4 | 5 |

**Brief Version of the Big Five Personality Test (NEO) translated version**

Instructions: The following are some statements about your feelings, attitudes, and behaviors. Please read each statement carefully and see if it applies to you. For each statement, indicate the extent to which you agree by marking the appropriate option (1 = Strongly Disagree; 2 = Disagree; 3 = Neutral; 4 = Agree; 5 = Strongly Agree). The questionnaire is anonymous, so please describe your feelings honestly. There are no right or wrong answers, and you don't need to spend too much time thinking about each question.

|  |  | Strongly Disagree | Disagree | Neutral | Agree | Strongly Agree |
| --- | --- | --- | --- | --- | --- | --- |
| 1 | I am not a person who worries a lot. | 1 | 2 | 3 | 4 | 5 |
| 2 | I like to be with many people. | 1 | 2 | 3 | 4 | 5 |
| 3 | I often enjoy immersing myself in fantasies and daydreams, exploring all possibilities, letting them spread and develop. | 1 | 2 | 3 | 4 | 5 |
| 4 | I try to be polite to everyone I meet. | 1 | 2 | 3 | 4 | 5 |
| 5 | I keep my belongings neat and clean. | 1 | 2 | 3 | 4 | 5 |
| 6 | I sometimes feel pain and indignation. | 1 | 2 | 3 | 4 | 5 |
| 7 | I love to laugh. | 1 | 2 | 3 | 4 | 5 |
| 8 | I find it interesting to develop new hobbies. | 1 | 2 | 3 | 4 | 5 |
| 9 | Sometimes I intimidate or deceive others to get them to do what I want. | 1 | 2 | 3 | 4 | 5 |
| 10 | I can manage my time well to get things done on time. | 1 | 2 | 3 | 4 | 5 |
| 11 | When I am under extreme stress, I sometimes feel like I am about to collapse. | 1 | 2 | 3 | 4 | 5 |
| 12 | I like jobs that I can complete alone without being disturbed by others. | 1 | 2 | 3 | 4 | 5 |
| 13 | I am fascinated by artistic and natural expressions. | 1 | 2 | 3 | 4 | 5 |
| 14 | Some people think I am selfish and self-centered. | 1 | 2 | 3 | 4 | 5 |
| 15 | I often encounter situations for which I am not fully prepared. | 1 | 2 | 3 | 4 | 5 |
| 16 | I rarely feel lonely or depressed. | 1 | 2 | 3 | 4 | 5 |
| 17 | I enjoy talking to people. | 1 | 2 | 3 | 4 | 5 |
| 18 | I think exposing students to controversial speakers will only confuse and mislead them. | 1 | 2 | 3 | 4 | 5 |
| 19 | I am a stubborn person. | 1 | 2 | 3 | 4 | 5 |
| 20 | I strive to complete all tasks assigned to me. | 1 | 2 | 3 | 4 | 5 |
| 21 | I often feel nervous and extremely uneasy. | 1 | 2 | 3 | 4 | 5 |
| 22 | I like to be in the crowd. | 1 | 2 | 3 | 4 | 5 |
| 23 | Poetry rarely affects me or even has no impact on me at all. | 1 | 2 | 3 | 4 | 5 |
| 24 | I know I am better than most people. | 1 | 2 | 3 | 4 | 5 |
| 25 | I have a series of clear goals and can achieve them step by step. | 1 | 2 | 3 | 4 | 5 |
| 26 | Sometimes I feel completely useless. | 1 | 2 | 3 | 4 | 5 |
| 27 | I avoid crowds. | 1 | 2 | 3 | 4 | 5 |
| 28 | Letting my mind wander freely is difficult for me. | 1 | 2 | 3 | 4 | 5 |
| 29 | If someone insults me, I try to forgive and forget. | 1 | 2 | 3 | 4 | 5 |
| 30 | It usually takes me a long time to settle down to work. | 1 | 2 | 3 | 4 | 5 |
| 31 | I rarely feel fear or anxiety. | 1 | 2 | 3 | 4 | 5 |
| 32 | I often feel energetic and full of vitality. | 1 | 2 | 3 | 4 | 5 |
| 33 | I rarely notice changes in my mood or feelings in different environments. | 1 | 2 | 3 | 4 | 5 |
| 34 | I tend to think the best of people. | 1 | 2 | 3 | 4 | 5 |
| 35 | I work hard to achieve my goals. | 1 | 2 | 3 | 4 | 5 |
| 36 | I often get angry at the way people treat me. | 1 | 2 | 3 | 4 | 5 |
| 37 | I am a happy and enthusiastic person. | 1 | 2 | 3 | 4 | 5 |
| 38 | I experience a variety of feelings and emotions. | 1 | 2 | 3 | 4 | 5 |
| 39 | Some people think I am cold and self-serving. | 1 | 2 | 3 | 4 | 5 |
| 40 | When I promise to do something, people believe I will see it through. | 1 | 2 | 3 | 4 | 5 |
| 41 | When things go wrong, I often feel discouraged and want to give up. | 1 | 2 | 3 | 4 | 5 |
| 42 | Talking with others does not give me much pleasure. | 1 | 2 | 3 | 4 | 5 |
| 43 | Reading a poem or viewing a piece of art sometimes stirs deep emotions in me. | 1 | 2 | 3 | 4 | 5 |
| 44 | My principles are practical rather than emotional. | 1 | 2 | 3 | 4 | 5 |
| 45 | I should be a reliable and trustworthy person, but sometimes I fall short. | 1 | 2 | 3 | 4 | 5 |
| 46 | I rarely feel sad or depressed. | 1 | 2 | 3 | 4 | 5 |
| 47 | My life is fast-paced. | 1 | 2 | 3 | 4 | 5 |
| 48 | I have little interest in pondering the nature of the universe or the human condition. | 1 | 2 | 3 | 4 | 5 |
| 49 | I usually try to think things through carefully. | 1 | 2 | 3 | 4 | 5 |
| 50 | I am productive and get things done on time and correctly. | 1 | 2 | 3 | 4 | 5 |
| 51 | I often feel helpless and want someone to solve my problems. | 1 | 2 | 3 | 4 | 5 |
| 52 | I am a very active person. | 1 | 2 | 3 | 4 | 5 |
| 53 | I am very curious about things that require brainpower. | 1 | 2 | 3 | 4 | 5 |
| 54 | If I don't like certain people, I let them know. | 1 | 2 | 3 | 4 | 5 |
| 55 | I seem to never get things organized. | 1 | 2 | 3 | 4 | 5 |
| 56 | Sometimes I feel so ashamed that I want to hide. | 1 | 2 | 3 | 4 | 5 |
| 57 | I prefer to act alone rather than lead others. | 1 | 2 | 3 | 4 | 5 |
| 58 | I am often interested in theories or abstract concepts. | 1 | 2 | 3 | 4 | 5 |
| 59 | When necessary, I will manipulate and control others to achieve my goals. | 1 | 2 | 3 | 4 | 5 |
| 60 | I strive for perfection in everything I do. | 1 | 2 | 3 | 4 | 5 |
