# Supplementary material for: Beyond metacognition: The dominant role of the general factor of personality in learning adaptation
Source: Heliyon. 2024 Jul 25;10(15):e35147. doi: 10.1016/j.heliyon.2024.e35147 (PMC11328064; doi:10.1016/j.heliyon.2024.e35147)
Supplement: Multimedia component 3 [file mmc3.docx]

**Questionnaire for Oral Metacognition**

Please read the following items carefully and then answer them according to your actual situation. This is not an exam, so there are not right or wrong answers. What is important is that every answer you choose could really represent your real feelings or opinions. Don’t hesitate on each one for a long time. Pay attention to the meaning of the numbers. Then fill in the number in the brackets before each item.

1. ( ) When I finish answering a question, I know whether I answer very well or badly.

2. ( ) I am accustomed to speaking when I see the question without taking time to plan language and thinking.

3. ( ) I can clearly sense the development of expression during oral English test.

4. ( ) I don’t form the habit of summarization when I finish speaking.

5. ( ) I am very clear whether my speed of gaining new knowledge is fast or slow.

6. ( ) I am not sure whether I can answer this question well unless I know what the tester expects.

7. ( ) I "trace" the process when I speak so that I can know my progresses or barriers.

8. ( ) I seldom try various methods to express.

9. ( ) I seldom feel I gain something new in the process of speaking after I finish it.

10. ( ) When I am held up in the oral test, I often feel at a loss and fidget.

11. ( ) I know my level of oral proficiency.

12. ( ) When I am held up in my thought, I usually cannot make in time adjustment and variation.

13. ( ) It is hard for me to not only speak but also pay attention to the process of speaking.

14. ( ) I know my study habit.

15. ( ) I never meet the questions about which I don’t know what to say and how to say.

16. ( ) I care much about whether my thinking is right instead of speaking only along one direction.

17. ( ) I don’t have accurate judgment about the difficulty of the question. I have either higher or lower estimation about them.

18. ( ) I am not accustomed to analyzing or improving my thinking and methods after I answer the questions.

19. ( ) When I take a step, I am not clear whether I better express myself.

20. ( ) When I am held up, I know I should change thinking but I don’t know how to do.

21. ( ) I am not sure how far away I am from the perfect expression in the oral English test.

22. ( ) I know my weak points in oral English.

23. ( ) Although I make a long speech, I am not sure whether it conforms to the questions.

24. ( ) I know whether I have a good memory.

25. ( ) I can quickly notice the incorrect thinking in oral English test.

26. ( ) I know my disparity when comparing with my classmates.

27. ( ) I know which part of content I have a good mastery.
